# Airway Epithelial Nucleotide Release Contributes to Mucociliary Clearance

**DOI:** 10.3390/life11050430

**Published:** 2021-05-11

**Authors:** Catharina van Heusden, Barbara R. Grubb, Brian Button, Eduardo R. Lazarowski

**Affiliations:** Marsico Lung Institute/Cystic Fibrosis Center, School of Medicine, University of North Carolina, Chapel Hill, NC 27599-7248, USA; c_vanheusden@med.unc.edu (C.v.H.); barbara_grubb@med.unc.edu (B.R.G.); brian_button@med.unc.edu (B.B.)

**Keywords:** ATP release, airway epithelia, mucociliary clearance, pannexin 1, vesicular nucleotide transporter

## Abstract

Mucociliary clearance (MCC) is a dominant component of pulmonary host defense. In health, the periciliary layer (PCL) is optimally hydrated, thus acting as an efficient lubricant layer over which the mucus layer moves by ciliary force. Airway surface dehydration and production of hyperconcentrated mucus is a common feature of chronic obstructive lung diseases such as cystic fibrosis (CF) and chronic bronchitis (CB). Mucus hydration is driven by electrolyte transport activities, which in turn are regulated by airway epithelial purinergic receptors. The activity of these receptors is controlled by the extracellular concentrations of ATP and its metabolite adenosine. Vesicular and conducted pathways contribute to ATP release from airway epithelial cells. In this study, we review the evidence leading to the identification of major components of these pathways: (a) the vesicular nucleotide transporter VNUT (the product of the SLC17A9 gene), the ATP transporter mediating ATP storage in (and release from) mucin granules and secretory vesicles; and (b) the ATP conduit pannexin 1 expressed in non-mucous airway epithelial cells. We further illustrate that ablation of pannexin 1 reduces, at least in part, airway surface liquid (ASL) volume production, ciliary beating, and MCC rates.

## 1. Introduction

The mucus gel layer covering the airway surface periciliary layer (PCL) traps inhaled materials and acts as a reservoir for water, buffering hydration of the PCL for needed cell surface lubrication and efficient ciliary beating [1]. When the hydration of the airway surface liquid (ASL) decreases, the mucus becomes hyperconcentrated, the PCL collapses, and the “thickened” mucus layer adheres to the cell surface, causing plaque/plug formation. Mucus stasis produces the airflow obstruction, infection, and inflammation characteristic of chronic obstructive lung diseases, including cystic fibrosis (CF) and chronic bronchitis (CB) [2,3,4,5]. Airway surface hydration is maintained by water fluxes mainly driven by active Cl^−^ and Na^+^ transport. Mutations in the CF transmembrane conductance regulator (CFTR) gene that result in abnormal CFTR Cl^−^ channel expression/activity are also associated with exacerbated Na^+^ absorption [6] and lead to ASL volume depletion in CF. The processes leading to mucus dehydration in non-CF lung diseases are incompletely described, but recent evidence suggests that prolonged cigarette smoke exposure reduces CFTR expression, induces CFTR internalization, and disrupts CFTR channel function, leading to ASL dehydration. Thus, cigarette smoke-induced CFTR dysfunction likely contributes to the onset of CB [7,8,9].

## 2. Purinergic Receptors Promote MCC Activities

**Figure 1:**
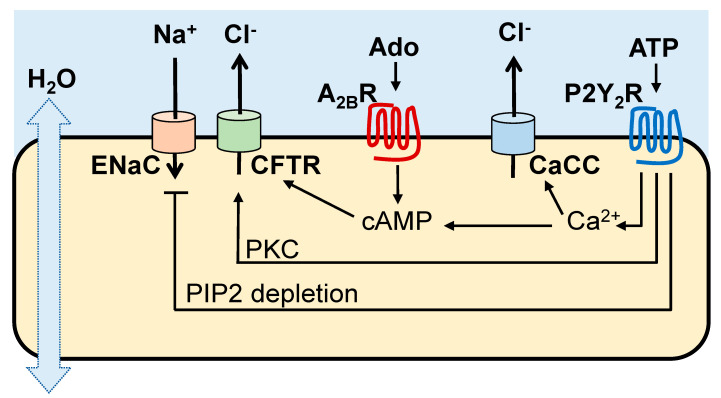
Purinergic regulation of mucus hydration and clearance The A_2B_R imparts cyclic AMP-regulated CFTR Cl^−^ channel activity, while the P2Y_2_R promotes Cl^−^ secretion via CaCC/TMEM16 and inhibits Na^+^ absorption. The P2Y_2_R also promotes CFTR-mediated Cl^−^ secretion PKC as well as Ca^2+^-promoted cyclic AMP formation.

Cl^−^ and Na^+^ transport activities in the airways are regulated by ASL concentrations of ATP and its metabolite adenosine acting on Gq-coupled P2Y_2_ receptors (P2Y_2_R) and Gs-coupled A_2B_R, respectively, expressed in non-mucous cells [10]. As summarized in Figure 1, the A_2B_R promotes cyclic AMP-regulated CFTR Cl^−^ secretion, whereas the P2Y_2_R promotes Cl^−^ secretion [via Ca^2+^ activated Cl^−^ channel (CaCC)/TMEM16 [11,12,13], protein kinase C (PKC)-mediated CFTR activation [14], and Ca^2+^-activated adenylyl cyclase I leading to cyclic AMP-mediated CFTR activation [15]]. In addition, the P2Y_2_R contributes to airway surface hydration by inhibiting Na^+^ absorption via phospholipase C-catalyzed depletion of phosphatidylinositol 4,5-bisphosphate [16,17,18].

In addition to ion transport regulation, activation of A_2B_ and P2Y_2_ receptors results in increased ciliary beating [19,20,21]. The P2Y_2_R also promotes mucin secretion from goblet cells [19].

## 3. Nucleotide Homeostasis in the Airways

Nucleotide release and hydrolysis rates must be finely balanced to control ATP and adenosine levels in ASL. Once released, ATP can interact with P2Y_2_R and be rapidly converted to adenosine by cell surface ectonucleotidases. Adenosine accumulates in ASL at levels capable of promoting A_2B_R/CFTR-dependent water transport in normal cells [22]. Due to the CFTR defect, the A_2B_R/cyclic AMP/CFTR mechanism is impaired in CF and, likely, in cigarette smoke-induced CB. Intriguingly, due to hydrolysis, ATP steady state levels are suboptimal to promote P2Y_2_R-mediated fluid secretion and, therefore, the CFTR-independent mechanism of airway hydration downstream of P2Y_2_R does not optimally hydrate CFTR-deficient epithelia [23].

Initial evidence for the purinergic control of ion/water secretion emerged from studies illustrating that: (a) baseline CFTR activity in Calu-3 lung epithelial cells was blocked by A_2B_R antagonists [24]; (b) apical addition of adenosine deaminase to primary cultures of naïve human bronchial epithelial (HBE) cells resulted in a CF-like ASL volume depletion [22]; and (c) the ASL height measured in CF HBE cells subjected to shear stress-promoted ATP release is markedly reduced following the addition of the ATPase apyrase [25]. Button and co-workers demonstrated that the hydration status of mucus correlated directly with the luminal rates of ATP release [26].

Relevant to CF, substantial literature, including our own studies, indicates that airway epithelial ATP release rates are not reduced in CF [22,25,27,28,29,30]. Notably, ATP release increased in HBE cells exposed to inflammatory stresses relevant to CF [27]. Furthermore, total nucleotide levels, in particular ADP and AMP, were found markedly elevated in CF lung secretions while ATP levels were reduced concomitantly with increased ATPase activity [23]. ATP concentrations also were reduced in CB lung secretions due to increased ATP hydrolysis activity [31]. These observations suggest that mucus dehydration and inflammation in CF and CB airways are associated with increased ATP metabolism leading to reduced ASL ATP levels [23,31]. Therefore, inhibition of ATP hydrolysis in CF and CB could be an approach to increase steady-state ATP levels on airway surfaces to restore airway hydration via P2Y_2_R-promoted activation of Cl^−^ secretion and inhibition of Na^+^ absorption. The recent identification of the polyoxometalate POM-5 as a potent and effective inhibitor of ATPase activities in airway epithelial cells and lung secretions [23] provided a useful tool to test this hypothesis. As we have recently shown, administration of POM-5 to CF HBE cells results in sustained, increased steady-state ATP levels in airway surfaces and enhanced ASL volume production [23].

## 4. Vesicular and Conductive Pathways of Airway Epithelial Nucleotide Release

Given the important role of released nucleotides in the control of airway surface hydration and mucus clearance, the mechanisms of nucleotide release by airway epithelia have been intensively investigated. Airway epithelial cells were initially shown to release ATP acutely, in response to mechanical stimuli, such as plate tilting or touching of the epithelium [28], and a medium change [30]. It has since been well-established that epithelial cells release discrete amounts of ATP constitutively [22,32] and that enhanced ATP release occurs following pharmacological challenges [33,34] or controlled mechanical stimuli, such as hypotonic cell swelling (16,50) or phasic motion that mimics the shear stress that is associated with normal tidal breathing (51). The diversity of conditions in which airway epithelial nucleotide release was observed suggested that different mechanisms (perhaps cell- and stimulus-specific) are involved [25,27,32,33,34,35,36,37,38,39]. For example, the mechanical strain exerted on the cilia via interaction with the overlying mucus layer promotes ciliated cell ATP release [26].

Non-lytic mechanisms for ATP release may include regulated exocytosis, which typically requires intracellular Ca^2+^_,_ and/or conductive/transporter pathways [40]. Primary cultures of naïve HBE cells, which are dominated by ciliated cells, exhibited robust release of ATP upon hypotonicity-induced cell swelling, and this release was, largely, not affected by chelation of the intracellular Ca^2+^ or by pharmacological inhibition of the secretory pathway [27,35,37], suggesting a conductive mechanism. However, HBE cells exposed to inflammatory challenges that promote goblet cell hyperplasia exhibited enhanced hypotonicity-induced ATP release, which was prevented by intracellular Ca^2+^ chelation and by reagents that disrupt vesicle trafficking/exocytosis [27,35]. Furthermore, agents that increase intracellular Ca^2+^ in HBE cells, such as ionomycin and UTP, caused a minor release of adenine nucleotides and mucins in naïve cultures, but ionomycin- and UTP-promoted ATP release and mucin secretion increased markedly in goblet cell-hyperplasic cultures [35]. Studies with goblet cell-rich Calu-3 cells demonstrated that mucin secreting cells release nucleotides as co-cargo-molecules from mucin-containing granules [34,36].

**Figure 2:**
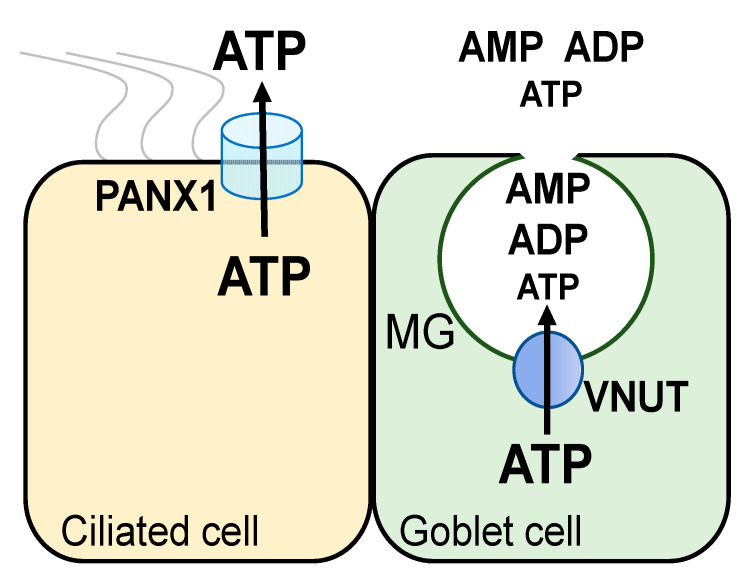
ATP release pathways in airway epithelia. Cytosolic ATP is released from ciliated cells via the plasma membrane channel PANX1. VNUT transports cytosolic ATP into Goblet cell mucin granules (MG). ATP and its metabolites within MG are secreted concomitantly with mucins.

Thus, functional data suggested that conducted and vesicular pathways contribute to ATP release from airway epithelial ciliated and goblet cells, respectively. Major components of these pathways have been identified at the molecular label: (a) pannexin 1 is an ATP release conduit expressed in non-mucous airway epithelial cells [41,42]; and (b) the vesicular nucleotide transporter VNUT [the product of the *SLC17A9* gene [43]] is the ATP transporter mediating ATP storage in (and release from) mucin granules and secretory vesicles [44] (Figure 2).

## 5. VNUT Mediates ATP Release from Mucin Granules and Vesicles

Our initial studies with goblet cell-rich airway epithelia established an association between nucleotide release and mucin secretion [35,36]. Calu-3 cells, a lung epithelial cell line comprised by a mixed population of non-mucous and mucin granule-rich (goblet) cells [36], exhibit both pannexin 1-mediated ATP release in response to cell swelling [41] and Ca^2+^ (ionomycin)-regulated vesicular release of nucleotides that correlates with mucin secretion [36]. Furthermore, the potent mucin secretagogue thrombin promoted robust nucleotide release in Calu-3 cells after complete inhibition of pannexin 1 [34]. Strikingly, ADP and AMP were the most abundant species accumulating in thrombin-stimulated Calu-3 cells, following pannexin inhibition. The data suggested that mucin granules store (and release) nucleotides. Analysis of the nucleotide composition in mucin granules isolated from Calu-3 cells supported this hypothesis. Notably, ADP, AMP, and ATP represented 60%, 30%, and 10% of the intragranular nucleotide pool, respectively [34], supporting the notion that ADP and AMP are the predominant nucleotide species released with mucin granules.

The identification by Moriyama and co-workers of SLC17A9/VNUT as the nucleotide transporter that transfers cytosolic ATP into secretory granules [43] provided a tool to investigate the association of mucin secretion and nucleotide release. VNUT mRNA was amplified in Calu-3 cells and strong VNUT immunoreactivity was observed in these cells [44]. Ca^2+^-regulated nucleotide release from Calu-3 cells was blunted after treatment with inhibitors of the secretory pathway and by downregulation of VNUT by shRNA [36,44]. Calu-3 cell fractionation yielded a VNUT immunoreactivity-rich fraction that sedimented with mucin granules. The relative distribution of ADP, AMP, and ATP within mucin granules was similar in control and VNUT shRNA-treated cells, but the total nucleotide pool was markedly reduced following VNUT knockdown [44]. This observation is consistent with the notion that VNUT transports ATP into mucin granules, but ATP is rapidly metabolized within the granular compartment [34,44] (Figure 2). Release of predominantly ADP and AMP from mucin granules minimizes autocrine, P2Y_2_R-mediated feedback for mucin secretion. Importantly, released AMP and ADP provide a source for adenosine formation leading to paracrine regulation of the ion/water transport activities needed for the hydration of newly released mucins.

In addition to mucin granules, VNUT immunoreactivity was observed in lysosome-rich and endoplasmic reticulum/Golgi-rich fractions isolated from Calu-3 cells [44]. Furthermore, confocal microscopy analysis of Calu-3 cells transfected with Myc-tagged VNUT revealed strong Myc immunoreactivity that co-localized with the mucin granule marker MUC5AC as well as vesicular compartments that stained negative for MUC5AC [44]. Our studies with inflamed airway epithelial cells suggest that a vesicular ATP pool can be released from cells independently from mucins. HBE cells exposed for two days to SMM (sterile supernatant from mucopurulent CF lung secretions) exhibited increased hypotonicity-promoted ATP release that was independent of pannexin 1 activation, was blocked by inhibitors of the secretory pathway, and was associated with increased VNUT expression, but was not accompanied by mucin secretion [27]. In line with these data, vesicular nucleotide release has been reported with cells lacking biochemically or morphologically defined secretory granules, e.g., lymphocytes [45], rat hepatoma cells [46], cholangiocytes [47], and lung carcinoma A549 cells [48,49,50].

Collectively, these observations suggest that VNUT transports ATP into (a) mucin granules in goblet cells, contributing to nucleotide release in mucin secreting cells, and (b) an unidentified vesicular compartment competent for regulated exocytosis in inflamed airway epithelia.

## 6. Pannexin 1 Mediated-ATP Release

The report by Dahl and co-workers that pannexin 1 acted as a plasma membrane ATP channel when overexpressed in *Xenopus* oocytes [51] triggered major interest in assessing the involvement of this channel in the release of ATP from mammalian cells, including airway epithelial cells. In 2009, Ransford et al. reported that ATP release from hypotonically swollen HBE cells was markedly inhibited by pannexin channel blockers or by knocking down pannexin 1 via shRNA [42]. Confocal images revealed pannexin 1 immunoreactivity at the apical membrane of most ciliated cells (the most abundant cell type in these cultures) although expression was not limited to these cells [42]. Simultaneously, we reported that activation of protease-activated receptors (PAR) in HBE cells resulted in enhanced release of ATP and enhanced uptake of the pannexin 1 permeant dye propidium iodide, and these responses were inhibited by the pannexin 1 blocker carbenoxolone [33]. Unambiguous assessment of the contribution of pannexin 1 to airway epithelial ATP release was subsequently obtained in our lab, using pannexin 1 knockout (*Panx1* KO) mice. Utilizing a perfusion approach to assess ATP levels in tracheal luminal secretions under controlled flow conditions, we reported that ATP release from wild-type (WT) tracheas increased up to six-fold following a brief exposure to hypotonicity. In contrast, *Panx1* KO animals exhibited impaired hypotonicity-evoked ATP release, and similar data were obtained with tracheal epithelial cell cultures from these mice [41]. In line with our studies, Workman et al. have recently illustrated that mechanical stimulation (via air puffs) of cultured WT murine nasal epithelial cells resulted in robust ATP release, which was markedly attenuated by the pannexin 1 inhibitor carbenoxolone and was greatly reduced (although not abolished) in cells from *Panx1* KO mice [52].

It is worth noting that ATP release conductive pathways additional to pannexin 1 have been described. For example, in the above-cited work, Workman and collaborators reported that, similar to *Panx1* KO cells, cells from mice lacking CALHM 1 [Ca^2+^ homeostasis modulator 1] exhibited reduced air puff-promoted ATP release. The authors concluded that pannexin 1 and CAHLM1 play complementary roles regulating ATP release in nasal epithelia [52]. CALHM 1 was initially describe as an ATP channel mediating taste-evoked ATP release from taste buds [53], but its expression in lower airway epithelia remains to be investigated. It has been recently shown that overexpression of ABCG1 (ABC subfamily G member 1) in HEK-293 cells confers enhanced hypotonicity-induced ATP release through volume-regulated anion channels (VRACs) [54]. However, the contribution of this pathway to airway epithelial ATP release is not known.

## 7. Pannexin 1 KO Mice Exhibit ASL Dehydration and Deficient Mucociliary Clearance

**Figure 3:**
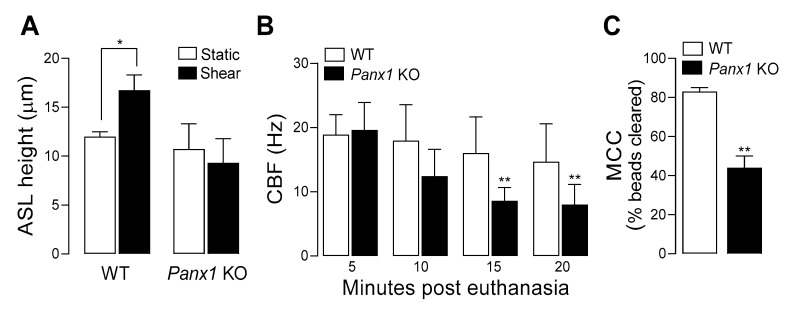
Pannexin 1 deletion reduces MCC activities. (**A**) ASL height was measured in MTE cells from WT and Panx1 KO mice, as described in Methods. Twelve measurements were performed per culture and 4 cultures were used per condition. The data are representative of two independent experiments; mean ± SD. (*) *p* < 0.05, Student’s *t*-test (Sigma Plot). (**B**): CBF was assessed in tracheas in situ after retracting the muscle and connective tissue. Exposed tracheas were lit with a DC red light and CBF was recorded under a dissecting microscope outfitted with a digital camera interfaced with Basler software; mean ± SD, n = 4–6. (**) *p* < 0.01, two-way ANOVA (Sigma Plot). (**C**): MCC was measured in 10 WT and 11 Panx1 KO mouse tracheas, in vivo. (**) *p* < 0.01, Student’s *t*-test.

Capitalizing on the availability of *Panx1* KO mice, we investigated the contribution of pannexin 1 to mucociliary clearance (MCC) activities. First, we evaluated the role of pannexin 1 in the regulation of airway surface hydration. ASL height was assessed in primary cultures of murine tracheal epithelial (MTE) cells, as an index of ASL volume production [26]. Under resting conditions, ASL height, as surrogate of the hydration status of the airway luminal surface [55] was similar in WT and *Panx1* KO cell cultures. However, when MTE cells were subjected to oscillatory shear stress [which mimics the phasic mechanical motion of the lung in vivo and results in increased ATP release [25,38,39]] ASL height increased in WT cells, but not in cells from *Panx1* KO mice (Figure 3A). These observations suggest that ASL height in static murine airway epithelia is not controlled by pannexin 1, but pannexin 1 promotes airway surface hydration under conditions relevant to the mechanical stresses exerted in normal lung physiology [25]. Ciliary beat frequency (CBF) and mucociliary transport rates were subsequently measured in murine tracheas in situ. CBF remained unchanged over time in WT mice but was markedly and significantly lower in *Panx1* KO mice after 15- and 20-min post euthanasia (Figure 3B). Lastly, utilizing a fluorescence micro-bead clearance assay previously described [56], MCC rates were found to be ~50% reduced in *Panx1* KO mice, compared to WT mouse (Figure 3C).

Collectively, these observations suggest that pannexin 1-mediated ATP release promotes airway surface hydration and ciliary beating, thus contributing to MCC activities in normal epithelia.

## 8. Summary and Conclusions

ATP and its metabolite adenosine present within the ASL, via activation of airway epithelial purinergic receptors, regulate multiple components of the MCC. Major pathways of nucleotide release and metabolism have been identified and their contribution to MCC activities is beginning to be understood. ATP is released onto the luminal surface from: (a) ciliated cells via the apical membrane channel pannexin 1; and (b) goblet cell mucin granules and secretory vesicles via VNUT-mediated transport. Released ATP interacts with the P2Y_2_R but is also rapidly converted to adenosine, which, in turn, promotes activation of the A_2B_R. Both the P2Y_2_R and the A_2B_R regulate ASL volume production and ciliary beating. Thus, decreased ATP release is predicted to result in defective MCC. Indeed, in normal, ciliated cell-dominated airway epithelia, a reduction in ATP released via ablation of pannexin 1 reduced, at least in part, ASL volume regulation, ciliary beating, and MCC rates. However, mucus hyperproduction and inflammation are associated with increased VNUT-mediated nucleotide release, suggesting that secretion of nucleotides from mucin granules and vesicles drives the hydration of newly secreted mucins. The extent to which pannexin 1- and VNUT-mediated nucleotide release contributes to mucous hydration in vivo in healthy and diseased airways is intensively investigated.

Due to the CFTR defect, the A_2B_R/cyclic AMP/CFTR-dependent mechanism of fluid secretion is impaired in CF and likely in cigarette smoke-induced CB. Intriguingly, due to hydrolysis, ATP steady-state levels are suboptimal to promote P2Y_2_R-mediated water secretion and, therefore, the CFTR-independent mechanism of airway hydration downstream of P2Y_2_R does not optimally compensate for the CFTR defect. Therefore, reducing the rates of hydrolysis of released ATP is predicted to facilitate airway surface hydration in CFTR-deficient airways.

## 9. Methods

Wild-type and *Panx1* KO mice were on C57BL/6 background, as previously described [41]. Mice 4 months of age and both genders were used. Mice were housed in individually ventilated micro-isolator cages, in a specific pathogen-free facility maintained at the University of North Carolina at Chapel Hill, on a 12-h day/night cycle. They were fed a regular chow diet and given water ad libitum.

MTE cells from WT and *Panx1* KO mice. Cells were harvested from tracheas from 6–8 WT and Panx1 KO mice and grown to confluence in ASL for 10 days, as described (39). ASL height was measure by confocal microscopy, as in [26]. For these studies, oscillatory shear stress was applied at 0.5 dynes/cm at 14 cycles per minute, as previously described (25).

CBF was measured in situ in the closed trachea, immediately after exsanguination of the mouse anesthetized with 3% isoflurane. For the tracheal preparation, the skin was opened, the muscle and connective tissue overlying the trachea were retracted and the exposed trachea was covered with a piece of plastic wrap dipped in water equilibrated mineral oil. This preparation was immediately placed under a dissecting scope (10× magnification) outfitted with a digital camera (Basler, Germany) interfaced with Basler software. The preparation was lighted with a DC red light allowing light reflected from the beating cilia to be easily seen. The temperature of the preparation was closely monitored using a temperature microprobe (T Type insect probe, Physitemp Inst., Clifton, NJ, USA) placed alongside the trachea. The output from the temperature probe was displayed digitally on a Physitemp TCAT-2ac Controller and a small ceramic heater (Wuhostam, Shenzhen, China) positioned close to the preparation was used to maintain the temperature of the preparation at 37 (±0.1) °C. Preparations were placed on an air table (TMC, Peabody, MA, USA) to minimize vibrations that interfered with the CBF measurements. It took approximately 3 min from the time of euthanasia until the preparation was under the scope and data acquisition commenced. CBF was measured for a 2 s period at the indicated times after euthanasia. The data were collected at 100 frames/second and analyzed using SAVA software. CBF is reported as the mean of values from the three time points for each mouse.

MCC activities in the trachea were assessed using the fluorescence micro-bead clearance assay previously described [56].

## Data Availability

The data presented in this study are available in the article.
